# Population Pharmacokinetics of Isavuconazole from Phase 1 and Phase 3 (SECURE) Trials in Adults and Target Attainment in Patients with Invasive Infections Due to Aspergillus and Other Filamentous Fungi

**DOI:** 10.1128/AAC.02819-15

**Published:** 2016-08-22

**Authors:** Amit Desai, Laura Kovanda, Donna Kowalski, Qiaoyang Lu, Robert Townsend, Peter L. Bonate

**Affiliations:** Astellas Pharma Global Development, Inc., Northbrook, Illinois, USA

## Abstract

Isavuconazole, the active moiety of the water-soluble prodrug isavuconazonium sulfate, is a triazole antifungal agent used for the treatment of invasive fungal infections. The objective of this analysis was to develop a population pharmacokinetic (PPK) model to identify covariates that affect isavuconazole pharmacokinetics and to determine the probability of target attainment (PTA) for invasive aspergillosis patients. Data from nine phase 1 studies and one phase 3 clinical trial (SECURE) were pooled to develop the PPK model (NONMEM, version 7.2). Stepwise covariate modeling was performed in Perl-speaks-NONMEM, version 3.7.6. The area under the curve (AUC) at steady state was calculated for 5,000 patients by using Monte Carlo simulations. The PTA using the estimated pharmacodynamic (PD) target value (total AUC/MIC ratio) estimated from *in vivo* PD studies of invasive aspergillosis over a range of MIC values was calculated using simulated patient AUC values. A two-compartment model with a Weibull absorption function and a first-order elimination process adequately described plasma isavuconazole concentrations. The mean estimate for isavuconazole clearance was 2.360 liters/h (percent coefficient of variation [%CV], 34%), and the mean AUC from 0 to 24 h (AUC_0–24_) was ∼100 mg·h/liter. Clearance was approximately 36% lower in Asians than in Caucasians. The PTA calculated over a range of MIC values by use of the nonneutropenic murine efficacy index corresponding to 90% survival indicated that adequate isavuconazole exposures were achieved in >90% of simulated patients to treat infections with MICs up to and including 1 mg/liter according to European Committee on Antimicrobial Susceptibility Testing methodology and in >90% of simulated patients for infections with MICs up to and including 0.5 mg/liter according to Clinical and Laboratory Standards Institute methodology. The highest MIC result for PTA was the same for Caucasian and Asian patients.

## INTRODUCTION

*Aspergillus* spp. and Candida spp. are common causes of invasive fungal infections (IFIs) in immunocompromised patients (1, 2). IFIs are associated with significant morbidity and mortality in this population (3, 4). Current therapeutic options for the treatment of IFIs, such as voriconazole, posaconazole, and itraconazole, are somewhat limited; thus, the development of a new antifungal agent would provide an alternative to existing therapies.

Isavuconazonium sulfate is a water-soluble triazole antifungal prodrug that is rapidly hydrolyzed by esterases to the active moiety, isavuconazole, and an inactive prodrug cleavage product (5). Isavuconazonium sulfate is available in oral (p.o.) and intravenous (i.v.) formulations. Isavuconazole's mechanism of action is inhibition of lanosterol 14α-demethylase, a microsomal P450 (P450_14DM_) enzyme essential for ergosterol biosynthesis in fungi (6). Previous analyses in healthy subjects have shown isavuconazole to have a volume of distribution in the range of 308.0 to 542.0 liters, a total systemic clearance (CL) of 2.4 to 4.1 liters/h, nearly complete bioavailability, and a half-life of 84.5 to 117.0 h (7).

Based on studies conducted in animal models and *in vitro*, isavuconazole has proven activity against clinically relevant fungi, including Candida spp., Aspergillus spp., Cryptococcus spp., and Mucorales organisms (8 – 15). In addition, as confirmed by phase 3 clinical trial (SECURE) data, isavuconazole has demonstrated noninferiority to voriconazole for the primary treatment of invasive mold disease caused by invasive aspergillosis (16), and it showed successful outcomes for patients with mucormycosis (17). Isavuconazonium sulfate has been approved by the U.S. Food and Drug Administration for the treatment of adults with invasive aspergillosis and invasive mucormycosis (18) and by the European Medicines Agency for the treatment of invasive aspergillosis and mucormycosis in cases where amphotericin B is inappropriate (19).

The aim of the present analysis was to develop a population pharmacokinetic (PPK) model for adults by using data pooled from healthy volunteers who participated in nine phase 1 studies and from patients who were enrolled in the SECURE clinical trial of IFIs caused by Aspergillus spp. and other filamentous fungi. The effects of various covariates were analyzed to determine their influence on the pharmacokinetics (PK) of isavuconazole and to determine if there were any differences in PK between healthy subjects and patients with IFIs. The secondary aim of the analysis was to determine the probability of achieving the pharmacokinetic-pharmacodynamic (PK-PD) target value (area under the curve [AUC]/MIC) after administration of a clinical dosing regimen over a range of MIC values by using Monte Carlo simulations.

## MATERIALS AND METHODS

### Subjects and patients.

In the phase 1 studies, the prodrug isavuconazonium sulfate was administered as either a single dose or multiple doses and administered either p.o. or as an i.v. infusion (1 h) to deliver 40 mg to 400 mg isavuconazole. In the SECURE clinical trial, a loading dose of 372 mg isavuconazonium sulfate (equivalent to 200 mg isavuconazole) was administered i.v. every 8 h for 6 doses (i.e., days 1 and 2), followed by a maintenance dose of 372 mg isavuconazonium sulfate (equivalent to 200 mg isavuconazole) administered once daily, p.o. or i.v., from day 3 to the end of treatment. A total of 421 individuals provided 6,363 drug concentration values (189 healthy subjects provided 5,828 drug concentration records, and 232 patients provided 535 drug concentration records) for modeling. None of the drug concentrations in the patient population had values below the quantification limit (BQL). BQL values for the healthy subject population comprised less than 5% of the total data and were therefore eliminated. The number of PK samples varied for phase 3 patients, depending on the duration of drug administration and the ability to collect PK samples. Also, only data for which dosing date and time and sampling date and time were recorded were included in the data set. Data used for modeling are summarized in Table 1.

**TABLE 1:** Description of data used for modeling^*a*^

Study no.	Design and objective (reference)	Phase	Isavuconazole dose(s) (mg)	Route	No. of subjects	Population	PK sampling times (h)
1	Double-blind, randomized, placebo-controlled, single-ascending-dose study (5)	1	100, 200, 400	p.o.	15	Healthy males	Predose, 0.25, 0.5, 0.75, 1, 1.25, 1.5, 2, 3, 4, 6, 8, 10, 12, 14, 16, 24, 36, 48, 60, 72, 96, 120, 144, 168, 192, 216, 240, 264, 288
2	Double-blind, randomized, placebo-controlled, single-ascending-dose study (5)	1	40, 80, 160	i.v.	18	Healthy males	Predose, 0.25, 0.5, 0.75, 1, 1.25, 1.5, 2, 3, 4, 6, 8, 10, 12, 14, 16, 24, 36, 48, 60, 72, 96, 120, 144, 168, 192, 216, 240, 264, 288
3	Double-blind, randomized, placebo-controlled, multiple-ascending-dose study (7)	1	50/100 p.o., 40/80 i.v.	p.o. and i.v. for 21 days	23	Healthy males	Predose, 0.25, 0.5, 0.75, 1, 1.25, 1.5, 2, 3, 4, 6, 8, 10, 12, 14, 16, 24, 36, 48, 60, 72, 96, 120, 240, 360, 480
4	Single-dose, open-label, parallel study to evaluate influence of hepatic impairment on PK of isavuconazole (27)	1	100	p.o. and i.v.	16	Healthy males and females	Predose, 0.5, 1, 1.5, 2, 3, 4, 6, 8, 10, 24, 48, 72, 96, 120, 144, 168, 216, 288, 360, 432, 480
5	Single-dose, open-label, parallel study to evaluate influence of hepatic impairment on PK of isavuconazole	1	100	p.o. and i.v.	16	Healthy males and females	Predose, 0.5, 1, 1.5, 2, 3, 4, 6, 8, 10, 24, 48, 72, 96, 120, 144, 168, 216, 288, 360, 432, 480
6	Open-label, randomized, 2-treatment crossover study to investigate bioavailability after single oral and i.v. doses of isavuconazole	1	400	p.o. and i.v.	14	Healthy males	Predose, 0.25, 0.5, 0.75, 1, 1.5, 2, 3, 4, 5, 6, 8, 10, 12, 16, 24, 48, 72, 96, 144, 192, 240, 288, 336, 504, 672, 840
7	Open-label, mass balance study to evaluate the PK of isavuconazole after single oral dose	1	200	p.o.	7	Healthy males and females	Predose, 0.5, 1, 2, 3, 4, 6, 8, 10, 12, 24, 48, 72, 96, 120, 144, 168, 216, 264, 312, 360, 408, 460, 504
8	Open-label, 2-part, parallel group study to assess the effect of renal impairment on the PK of isavuconazole	1	200	i.v.	32	Healthy males and females	Predose, 1, 1.5, 2, 3, 4, 5, 6, 8, 12, 24, 36, 48, 72, 120, 168, 240, 288, 336
9	Open-label, parallel group, single-dose study to evaluate the PK profile of isavuconazole in healthy nonelderly and elderly male and female subjects	1	200	p.o.	48	Healthy and elderly males and females	Predose, 1, 1.5, 2, 3, 4, 5, 6, 8, 12, 24, 36, 48, 72, 120, 168, 240, 288, 336
10	Phase 3, double-blind, randomized study to evaluate safety and efficacy of isavuconazole vs voriconazole	3	200	i.v./p.o.	232	Isavuconazole patients	Trough concentrations on days 7, 14, and 42 and EOT PK profiling (predose, 1.5, 3, 4, 6, 12, and 24 h postdose for 20 patients on either day 7 or day 14)

a Abbreviations: EOT, end of treatment; i.v., intravenous; PK, pharmacokinetics; p.o., oral.

### Data analysis.

The population analyses were conducted via nonlinear mixed-effects modeling with the software program NONMEM, version 7.2 (GloboMax LLC, Hanover, MD), on a Hewlett-Packard model Z400 64-bit personal computer with Windows 7M and an Intel Xeon processor, using Pirana, version 2.7.0 (http://www.pirana-software.com). The first-order conditional estimation method in NONMEM was employed for all model runs. The interaction option was not employed because both the concentration data and residual error structure were log transformed. The ADVAN 4 TRANS 4 option in NONMEM was used for modeling purposes. Model selection was driven by the data and based on the following goodness-of-fit criteria: (i) visual inspection of diagnostic scatterplots (observed versus individual predicted concentrations and residuals/weighted residuals versus predicted concentrations or time); (ii) successful convergence of the minimization routine, with at least 2 significant digits in parameter estimates; (iii) precision of parameter estimates; and (iv) the minimum objective function value and number of estimated parameters.

### Structural pharmacokinetic model.

A variety of linear compartmental PK models were explored to describe isavuconazole concentration-time data. The base PPK model included two and three compartments, with either first-order absorption models or a Weibull absorption model. Absolute bioavailability (*F*) was fixed at 1, based on results from previous noncompartmental analyses of healthy subjects. All random effects were treated as log-normally (Ln) distributed. The Ln-Ln transformations of both the output equation in the model and the data were used to stabilize the residual variance. The residual variance was modeled as additive in nature. The models were coded using the NONMEM subroutines for prediction of PPK parameters.

### Pharmacokinetic model with covariates.

Following development of the base structural and statistical PPK model, a covariate analysis was conducted, and the best covariate model was selected. Stepwise covariate modeling was performed in Perl-speaks-NONMEM, version 3.7.6 (http://psn.sourceforge.net), by forward inclusion and backward elimination steps. In the forward inclusion step, covariates were added to the model one at a time, using a *P* value cutoff of <0.01 for entry into the model. In the backward elimination step, covariates were removed one at a time, using a *P* value cutoff of <0.001 for retention in the model. Stringent criteria were utilized to retain only clinically relevant covariates in the model (20, 21). The process was continued until all remaining covariates were significant. The covariates that were analyzed in the model are presented in Table 2; demographics and baseline characteristics of the healthy subjects and patients are summarized in Table 3.

**TABLE 2:** Covariates analyzed in the PPK model^*a*^

Type of covariate	Covariate
Continuous	wt (kg)
	BMI (kg/m^2^)
	ht (cm)
	Lean body mass (kg)
	Age (yr)
	Liver chemistry (ALT, AST, TBILI, ALB, ALKPHOS)
Categorical	Race (0 for predominantly Caucasians, 1 for Asians)
	Sex
	SP (dichotomized into healthy subjects [0] and patients [1])
	CONMEDS (CYP3A inhibitors; strong or weak/mild)

a Lean body mass was calculated based on the James formula (35). Abbreviations: ALB, albumin; ALKPHOS, alkaline phosphate; ALT, alanine transaminase; AST, aspartate aminotransferase; CONMEDS, concomitant medications; PPK, population pharmacokinetics; SP, subjects and patients; TBILI, total bilirubin concentration. CONMEDS were classified based on the information at http://medicine.iupui.edu/clinpharm/ddis/main-table.

**TABLE 3:** Demographics and baseline characteristics^*a*^

Baseline characteristic	Value	
	Healthy subjects (*n* = 189)	Patients with IFIs (*n* = 232)
Sex (no. [%])		
Male	140.0 (74.1)	132.0 (56.9)
Female	49.0 (25.9)	100.0 (43.1)
Race (no. [%])		
Predominantly Caucasian	175.0 (92.6)	193.0 (83.2)
Asian	14.0 (7.4)	39.0 (16.8)
Age (yr) (median [range])	43.0 (19.0–85.0)	54.0 (17.0–82.0)
ht (cm) (median [range])	175.0 (148.0–196.0)	168.4 (145.0–200.0)
wt (kg) (median [range])	77.8 (51.7–118.3)	67.0 (41.0–127.7)
BMI (kg/m^2^) (median [range])	25.7 (18.0–34.7)	23.6 (13.9–41.1)
Lean body mass (kg) (median [range])	58.4 (37.7–77.7)	50.8 (32.5–82.6)

a Lean body mass was calculated based on the James formula (35). The predominantly Caucasian population consisted of one African American patient, five patients classified in other race categories, and the remaining patients classified as Caucasian. IFIs, invasive fungal infections.

### Model validation.

For the base and best covariate models, population and individual PK parameters were estimated, and the precisions of the population model parameters (e.g., asymptotic standard errors or bootstrap 95% confidence intervals [CIs]) were generated. Nonparametric bootstrapping using 500 replications was used to provide validation of the model parameter estimates. Normalized prediction distribution errors (NPDE) were also plotted to evaluate the best model.

### Probability of target attainment (PTA).

As described by Drusano et al. (22), Monte Carlo simulations were performed using mean population estimates from the best covariate model. A total of 5,000 patients were simulated. The dosing regimen that was used for simulation was a loading dose of 372 mg isavuconazonium sulfate (equivalent to 200 mg isavuconazole) administered i.v. every 8 h for 6 doses (i.e., days 1 and 2) followed by a maintenance dose of 372 mg isavuconazonium sulfate (equivalent to 200 mg isavuconazole) administered once daily, p.o. or i.v., for 840 h. The AUC was calculated over the time interval of 840 to 864 h, which was at steady state considering the long half-life of the drug.

Any covariates found to be significant were randomly added from the available studies to the simulation data set. Experimental PD models were conducted to establish the exposure-response relationship associated with efficacy and to estimate the target exposure associated with the optimal exposure-response relationship (PD target). For isavuconazole, the PD index associated with efficacy in murine models of aspergillosis is the AUC/MIC ratio (23). The PD target (total drug AUC/MIC) for 50% survival estimated for the nonneutropenic murine model of disseminated aspergillosis is 50.4 as determined with MIC values tested via Clinical and Laboratory Standards Institute (CLSI) methodology and 24.7 as determined with MIC values tested via European Committee on Antimicrobial Susceptibility Testing (EUCAST) methodology (23). The PTA was calculated as the fraction of patients who attained the target over the range of MIC values studied (0.03 mg/liter to 8 mg/liter).

## RESULTS

### Base model for PPK analysis.

PPK analysis was used to determine the effects of covariates on the PK of isavuconazole. It was also used to determine the variability between patients with IFIs and to identify any differences in PK between healthy subjects and patients. A PPK model was developed using rich data from various phase 1 studies and predominantly trough levels from one phase 3 study. It was then utilized to perform Monte Carlo simulations and to determine the PTA over a range of MIC values.

The PK model development process resulted in a base model that included two compartments and a Weibull absorption function (24). The Weibull absorption function was chosen over a simple model with first-order absorption due to a change in the objective function value of >1,000 points, and also due to a better goodness of fit as assessed through plots (data not shown).

The following structural PK model described by three ordinary differential equations was used:

$$\frac{dA(1)}{dt}=-\text{WB}\times A(1)$$

$$\text{WB}=\text{KAMAX}\times [1-e^{\left(-(RA\times TAD{)}^{GAM}\right)}]$$

$$\frac{dA(2)}{dt}=\text{WB}\times A(1)-\frac{\text{CL}}{V}\times A(2)-K_{cp}\times A(2)+K_{pc}\times A(3)$$

$$\frac{dA(3)}{dt}=K_{cp}\times A(2)-K_{pc}\times A(3)$$

where *A*(1), *A*(2), and *A*(3) represent the amounts of isavuconazole in the gut, central, and peripheral compartments, *K*_cp_ and *K*_pc_ are the first-order intercompartmental rate constants, WB is the Weibull function, CL is the clearance, *V* is the volume of distribution, and TAD is the time after dose.

The model had interindividual variability on clearance (CL), the volume of distribution in the peripheral compartment (*V*_p_), intercompartmental clearance (*Q*), and the Weibull absorption parameters (RA and GAM1). The data for one patient who was considered an outlier were removed from the overall analysis due to an extremely low clearance value (0.2 liter/h). The model development procedure did not show interindividual variability to be statistically significant for any other parameter.

### Best model with covariates.

Following addition of covariates to the base model, the best full covariate model included effects of race on CL, body mass index (BMI), and the difference between subjects and patients (SP) on *V*_p_. Clearance values did not change over time and were similar between different dose groups. The covariate model is represented as follows: CL = θ_1_ (Caucasian) and θ_9_ (Asian) and *V*_p_ = θ_4,11_ × (1 + θ_10_) × (BMI − 24.80), where θ_4,11_ is for healthy subjects and patients.

**FIG 1:**
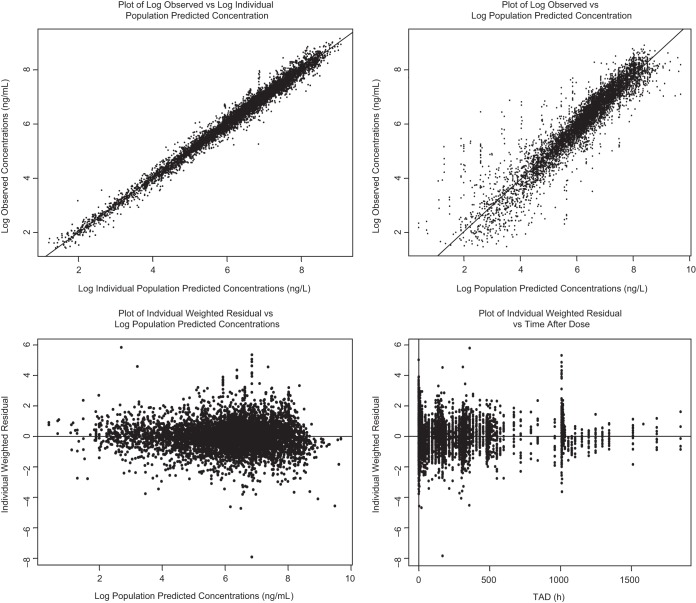
Goodness-of-fit plots for the best covariate model. TAD, time after dose.

The total AUC for individual subjects and patients was calculated using the standard formula (AUC = *F* × dose/CL; *F* = 1), based on the individual parameter estimates from the best model. Isavuconazole AUCs for healthy subjects and patients are presented in Table 4. Goodness-of-fit plots (Fig. 1) showed that the model was consistent with the observed data, no systematic bias was evident (data not shown), and no observable covariate trends remained within the full model. Parameter estimates are shown in Table 5.

**TABLE 4:** AUC values obtained using individual clearance values from the best model with covariates^*a*^

Parameter	Value		
	Healthy subjects	Patients with IFIs	Combined (healthy subjects + patients)
AUC (mg·h/liter)			
Mean (SD)	92.0 (31.6)	101.0 (56.0)	97.0 (48.0)
Median	88.3	89.6	88.3
Minimum	33.0	10.3	10.3
Maximum	244.7	343.5	343.5
%CV	34.4	55.4	48.3

a Abbreviations: AUC, area under the curve; CV, coefficient of variation; IFIs, invasive fungal infections; SD, standard deviation.

**TABLE 5:** Parameter estimates of the best covariate model^*a*^

Parameter	Units	Value	SE	% RSE	Bootstrap mean	Bootstrap 95% CI
θ_1_ (CL, Caucasian)	Liters/h	2.36	0.52	2.2	2.36	2.25–2.46
θ_2_ (*V*_1_)	Liters	49.10	1.58	3.2	49.27	46.09–52.15
θ_3_ (*Q*)	Liters/h	26.60	1.09	4.1	26.60	24.60–28.70
θ_4_ (*V*_p_)	Liters	417.0	18.50	4.4	418.09	381.52–453.00
Weibull absorption parameters						
θ_5_ (KAMAX)	h^−1^	1.08	0.091	8.4	1.10	0.89–1.27
θ_6_ (RA)	h^−1^	0.72	0.029	4.1	0.72	0.66–0.78
θ_7_ (GAM1)		4.88	0.239	4.9	4.87	4.41–5.35
Covariates						
θ_9_ (CL, Asian)	Liters/h	1.51	0.059	10	1.54	1.0–1.99
θ_10_ (BMI on *V*_p_)		0.060	0.006	9.5	0.064	0.051–0.076
θ_11_ (SP on *V*_p_)	Liters	260	0.031	2	259.4	217–302
Variability (%)						
CL (healthy subjects)		31.30	0.012	6	30.98	26.45–34.64
*V*_p_		31.78	0.138	7	31.62	27.01–35.77
RA		40.24	0.019	6	40.24	35.35–44.60
*Q*		49.09	0.037	8	49.09	41.35–55.76
GAM1		45.71	0.041	10	45.82	35.63–53.99
CL (patients)		62.44	0.066	8	62.44	51.08–73.21
Residual error (%)						
θ_8_ (*W*)		44.94	0.004	2	44.83	43.93–45.93

a Parameter values were rounded to the nearest decimal place. Abbreviations: BMI, body mass index; CL, clearance; KAMAX RA, and GAM1, Weibull absorption parameters; *Q*, intercompartmental clearance; RSE, relative standard error; SE, standard error; SP, difference between healthy subjects and patients; *V*_1_ and *V*_p_, volumes of distribution of the central compartment and peripheral compartment, respectively; *W*, weighting factor.

### Model validation.

**FIG 2:**
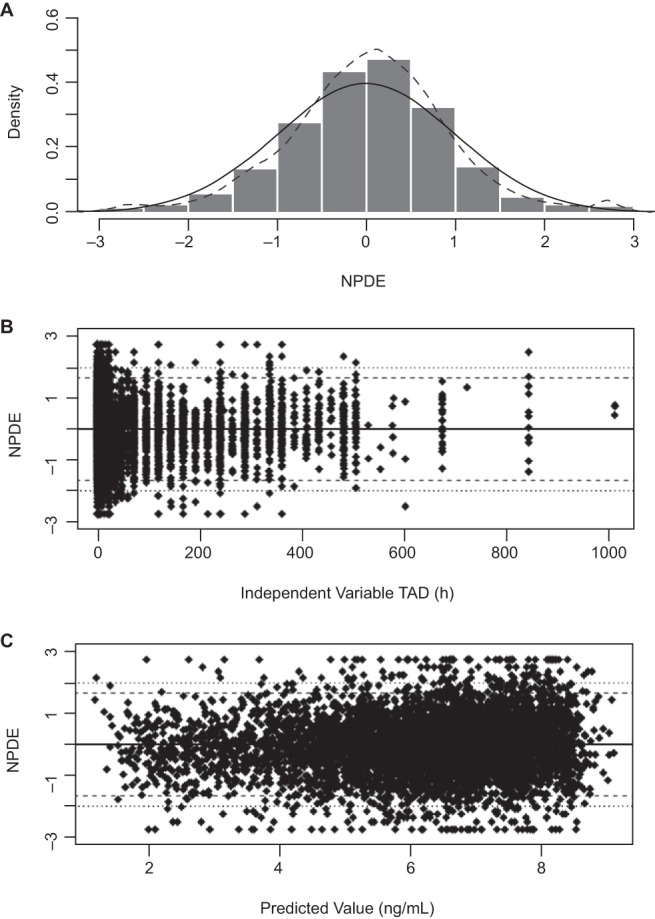
NPDE plots. NPDE, normalized prediction distribution error.

The results for 500 bootstrap replicates are summarized in Table 5. The mean estimates for all parameters from the bootstrapping procedure were very similar to the parameter estimates from the best covariate model. Only 13% of the bootstrapping runs failed. Failed runs during bootstrapping were excluded from calculations and are not reported. This indicated the adequacy and stability of the model and that the estimates for fixed and random effects were accurate. Plots of the NPDE demonstrated that the normality assumption was not rejected (Fig. 2A), and plots of NPDE versus time (independent variable) and predicted concentrations did not show any obvious trends (Fig. 2B and C).

### Probability of target attainment.

**FIG 3:**
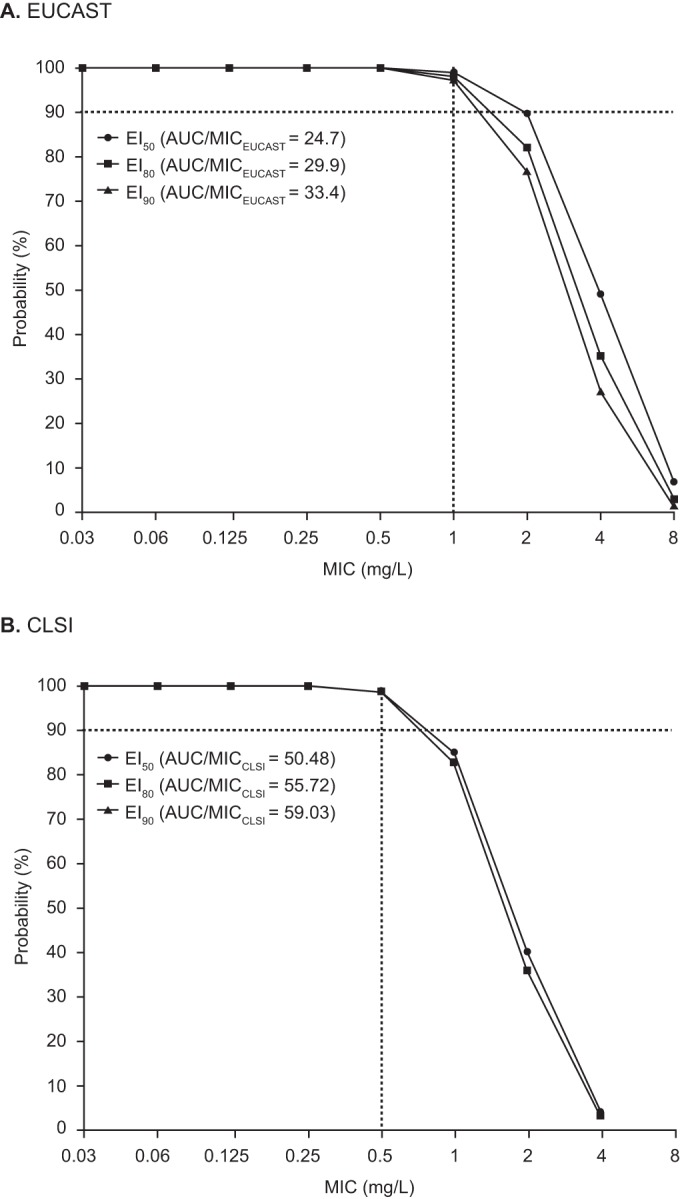
Probability of target attainment over a range of MIC values for EI_50_, EI_80_, and EI_90_. (A) EUCAST methodology; (B) CLSI methodology. For panel B, note that the EI_80_ and EI_90_ values overlap in the plot. AUC, area under the curve; CLSI, Clinical and Laboratory Standards Institute; EUCAST, European Committee on Antimicrobial Susceptibility Testing.

Overall, 5,000 patients were simulated using Monte Carlo simulation with the mean parameter estimates, including variability on patients, from the best model with covariates. BMI was randomly added based on values found in the National Health and Nutrition Examination Survey 2014 (NHANES; http://www.cdc.gov/nchs/nhanes.htm), with the difference in BMI ranges between Caucasian and Asian patients taken into consideration. The BMI range was 14 to 41 kg/m^2^ for Caucasian patients and 17 to 28 kg/m^2^ for Asian patients. All PD targets that were established from different experimental models were used in the PTA analysis. The results of PTA analysis performed for the nonneutropenic mouse model and for both CLSI and EUCAST methodologies are shown in Fig. 3. The AUC/MIC range was derived for each patient, and the percentages of patients above exposure index (EI) values of 50%, 80%, and 90% were plotted against the range of MIC values. The target values for EI_50_, EI_80_, and EI_90_ were 24.7, 29.8, and 33.3, respectively, for MICs determined according to EUCAST methodology and 50.4, 55.7, and 59.0, respectively, for MICs determined by CLSI methodology (23). These values indicate the exposures required for 50%, 80%, and 90% survival for all isolates. For all the PD target values, the PTA analysis demonstrated that >90% of the simulated patients would achieve adequate exposures to treat infections with MIC values of ≤1 mg/liter according to EUCAST methodology and that >90% of the simulated patients would achieve adequate exposures for treatment of infections with MICs of <1 mg/liter according to CLSI methodology.

## DISCUSSION

The PK parameters of isavuconazole were best described by a two-compartment model with a Weibull absorption function and linear elimination. For a typical patient, the Weibull function described the absorption phase adequately, with complete absorption at 2 to 3 h postdosing. All parameters in the model were precisely estimated, with percent relative standard errors of ≤10% for the fixed effects and ≤10% for the random effects (Table 5). The condition number of 41, which is far below the critical threshold of 1,000 (20), implies that the model was stable and was not overparameterized. None of the goodness-of-fit plots showed systematic bias, as all model diagnostics indicated an overall good fit of the model to the data. Internal validation to determine the best covariate model was performed using a nonparametric bootstrapping procedure. Bootstrapping resulted in a 95% CI for each fixed- and random-effect parameter that showed little bias from estimated values (Table 5). The robustness of the results was strengthened by the inclusion of data from phase 1 and phase 3 studies in the analysis.

Covariate analysis was performed to identify the influence of various demographic or baseline characteristics, if any, on the PK of isavuconazole. No difference in exposure was observed between healthy subjects and patients. Mean AUC values for healthy subjects and patients were 92 mg·h/liter and 101 mg·h/liter, respectively, with 55% between-subject variability observed for patients, compared to 36% for healthy subjects. The ages of the analysis population members ranged from 17 to 85 years; however, no difference in PK parameters or exposure was noted between young and elderly adults, indicating that age had no effect on isavuconazole PK. The 17-year-old patient was 20 days from turning 18 years old at the time of first dose administration and therefore was included in the study. The only statistically significant covariate on CL was race. The population mean estimate of CL was 2.360 liters/h, with Asians having an approximately 36% lower CL value (1.510 liters/h) than that of the predominantly Caucasian population (see Table 3 for a description of the group described as “predominantly Caucasian”). Due to lower CL values, Asians have higher exposures than Caucasians. The clearance value estimated for Caucasians (2.4 liters/h) was consistent with values observed in healthy subject studies (7). The exact reason for lower CL values in Asians has not been established. The potential contribution of either cytochrome P450 2D6 (CYP2D6) (25) or CYP2C19 (data on file at Astellas Pharma Global Development, Inc.) is unlikely, as isavuconazole is not a substrate for either isoenzyme (26). BMI was also a statistically insignificant covariate on CL, which eliminates differences in body mass as a contributing factor for the difference in CL between these two population groups. In addition, dose was not shown to be an influential covariate. Body weight was also a statistically insignificant covariate on any parameter of interest.

The best model was a two-compartment model with Weibull absorption and first-order elimination, indicating that exposure of isavuconazole increased proportionally with increasing doses. From the PPK model, it can be concluded that isavuconazole PK are linear and dose proportional, with variability in patients of around 60% (Table 5), which is useful for predicting plasma concentrations of isavuconazole in the patient population.

The mean terminal half-life of isavuconazole for the patient population was estimated to be 130 h, with a median of 110 h and 5th and 95th percentiles of 53.5 and 248.1 h, respectively, similar to the half-life observed for healthy subjects as analyzed by noncompartmental methods (27). Once-daily administration of 200 mg isavuconazole is sufficient to maintain isavuconazole concentrations to treat infections with MICs up to and including 1 mg/liter according to EUCAST methodology and for >90% of patients with infections with MICs up to and including 0.5 mg/liter according to CLSI methodology.

Two covariates were shown to significantly influence *V*_p_, namely, BMI and SP. There was a direct relationship between *V*_p_ and BMI, with an increased BMI leading to an increased *V*_p_. BMI values ranged from 13.89 kg/m^2^ to 41.18 kg/m^2^; 43 individuals had BMI values of >30 kg/m^2^ and were considered obese. *V*_p_ was greater in obese individuals than in nonobese individuals; however, there was no difference in exposure between these groups. *V*_p_ was also greater in patients (∼390 liters) than in healthy subjects (∼292 liters). The model estimated that the *V* at steady state was approximately 460 liters, which implies that the drug is distributed in various tissues in the body, although the exact distribution or distribution to any particular tissue is unknown. The covariate analysis did not reveal any clinical consequences, as exposures were not significantly affected by any particular demographic or baseline characteristic except for race.

The probability of PK-PD target attainment over a range of MIC values was determined for patients. The goal of PTA analysis was to determine the MIC at which at least 90% of the population achieved the PD target for the AUC/MIC value. A probability of ≥90% is recognized as an acceptable PD target by both EUCAST and CLSI (28, 29). Monte Carlo simulations were performed using the mean estimates from the best covariate model. Phase 3 trial isavuconazole dosage regimens were used to perform the simulations, and the total AUC from 0 to 24 h (AUC_0–24_) was calculated at steady state for the simulated patients. Total AUC was used instead of unbound AUC because protein binding for isavuconazole is >99% in humans (30) and protein binding levels between species (rats, mice, and humans) are very similar (data on file at Astellas Pharma Global Development, Inc.).

Experimental PD models were conducted to establish the exposure-response relationship associated with efficacy and to estimate the target exposure associated with the optimal exposure-response relationship (PD target). While several models have been used to assess the PD target in the setting of experimental invasive aspergillosis (23, 31, 32), the PD targets of 24.8 (EI_50_) for EUCAST methodology and 50.4 (EI_50_) for CLSI methodology (23) from the nonneutropenic mouse model were retained, as they provided results concordant with the results of the clinical trial and with the epidemiologic cutoff values (ECVs) for Aspergillus species. ECVs have been reported previously for isavuconazole and Aspergillus spp. (33, 34). For Aspergillus fumigatus, A. flavus, and A. terreus, the EUCAST and CLSI ECVs are 2 mg/liter and 1 mg/liter, respectively. In each of the PD models conducted, successful outcomes were demonstrated for Aspergillus species isolates with isavuconazole MIC values of up to 2 mg/liter and 4 mg/liter for the CLSI and EUCAST methodologies, respectively, regardless of the presence of CYP51A mutations (23, 31, 32). Figure 3 shows the PTA over a range of MIC values for EI_50_, EI_80_, and EI_90_ (effective PD indexes for 50%, 80%, and 90% survival of the nonneutropenic infected mice) for both the CLSI and EUCAST methodologies. The results showed that the clinical dosing regimen achieved exposures adequate to treat infections with MICs up to and including 1 mg/liter as determined by EUCAST methodology and close to 90% of infections for the CLSI EI_50_ target. We analyzed the concentration-time profile, AUC, and PTA separately for Asian patients and Caucasian patients, in contrast to analyzing them together; however, since the findings did not warrant dose adjustment, we have not presented these data separately.

In summary, a robust PPK model was developed using data from phase 1 and SECURE studies. The PPK model fit the data adequately. Isavuconazole showed dose-proportional PK, with no relevant PK differences between healthy subjects and patients with IFIs. The only clinically important covariate that affected isavuconazole exposure was a 40% difference in exposure between predominantly Caucasian patients and Asian patients, an effect whose mechanism has yet to be elucidated. The proposed clinical dosing regimen of a 372-mg isavuconazonium sulfate loading dose (equivalent to 200 mg of isavuconazole) every 8 h for 6 doses, p.o. or i.v., followed by maintenance doses of 372 mg isavuconazonium sulfate (equivalent to 200 mg of isavuconazole) once daily, p.o. or i.v., would adequately treat isolates with MICs up to and including 1 mg/liter according to EUCAST methodology and >90% of isolates with MICs up to and including 0.5 mg/liter according to CLSI methodology.
